# Further Observations on the Use of Pacemakers in Patients with Postural Orthostatic Tachycardia Syndrome with Demonstrated Asystole

**DOI:** 10.19102/icrm.2021.120307

**Published:** 2021-03-15

**Authors:** Khalil Kanjwal, Asim Kichloo, Rehana Qadir, Blair P. Grubb

**Affiliations:** ^1^Division of Cardiology, McLaren Greater Lansing Hospital, Michigan State University, Lansing, MI, USA; ^2^Division of Internal Medicine, Central Michigan University, Mount Pleasant, MI, USA; ^3^Division of Internal Medicine, McLaren Greater Lansing Hospital, Michigan State University, Lansing, MI, USA; ^4^Division of Cardiology, University of Toledo, Toledo, OH, USA

**Keywords:** Loop recorder, neurocardiogenic syncope, postural orthostatic tachycardia syndrome

## Abstract

A subgroup of postural orthostatic tachycardia syndrome (POTS) patients may also have features of neurocardiogenic syncope (NCS). Syncope and presyncope are predominant clinical features in this subgroup of patients. Asystole has been reported as the cause of some recurrent syncopal episodes following evaluation with an implantable loop recorder (ILR). We present our experience of pacing in a group of patients with POTS and NCS, which resulted in the complete elimination of syncope. We reviewed the charts of 500 patients at the University of Toledo Medical Center from 2003 to 2013 and identified 40 patients who were eligible for inclusion in this study. Patients were included in this study if they had clinical features of POTS and unusually frequent episodes of syncope. All study participants subsequently underwent ILR implantation. Forty patients, including 32 (80%) women, aged 33 ± 13 years were included in this study. All patients demonstrated prolonged asystole (> 6 seconds) or severe bradycardia (heart rate < 30 bpm) during their syncope. Ten patients demonstrated an asystole of more than 10 seconds and also had prolonged and convulsive syncope. All patients had abrupt syncope without any warning signs. All 40 patients underwent dual-chamber pacemaker implantation. Syncope was eliminated in all 40 patients following pacemaker implantation; however, they continued to experience orthostatic tachycardia. Our findings support that dual-chamber pacing may help to eliminate syncope in a subgroup of POTS patients with recurrent syncope and prolonged asystole on ILR.

## Introduction

A combined form of neurocardiogenic syncope (NCS) and postural orthostatic tachycardia syndrome (POTS) has been previously reported in a subgroup of patients.^1^ In these individuals, syncope and presyncope are common and predominant symptoms. These patients were found to have prolonged asystole as a mechanism of their syncope when evaluated by an implantable loop recorder (ILR).^2^

## Methods

This was a retrospective study approved by the institutional review board at the University of Toledo. We reviewed the charts of 700 POTS patients seen at our autonomic center at the University of Toledo from 2003 to 2013 and found 40 patients who were eligible for inclusion in this study. Due to the study’s retrospective nature, the need for informed consent was waived.

### Criterion for diagnosing orthostatic intolerance

Orthostatic intolerance (OI) is the umbrella term for a heterogeneous group of disorders of hemodynamic regulation characterized by excessive pooling of blood in the dependent areas of the body during upright posture, resulting in insufficient cerebral perfusion, which triggers symptoms typically relieved by recumbency. Both POTS and NCS patients have OI.

### Criterion for diagnosing postural orthostatic tachycardia syndrome

POTS was defined for this study as having continuous symptoms of OI (> 6 months’ duration) accompanied by a heart rate increase of at least 30 bpm (or a rate that exceeds 120 bpm) observed during the first 10 minutes of upright posture or the head-up tilt test (HUTT) occurring in the absence of other chronic debilitating disorders.^1,3^ Symptoms include fatigue, orthostatic palpitations, exercise intolerance, light-headedness, diminished concentration, headache, near-syncope, and syncope. In a retrospective chart review, we collected data including demographic information, presenting symptoms, laboratory data, tilt-table response, and treatment outcomes.

### Criterion for diagnosing neurocardiogenic syncope

NCS was defined as episodic syncope (transient loss of consciousness) with spontaneous recovery. The criterion for the diagnosis of NCS included a HUTT response consistent with NCS [a sudden decrease in heart rate and/or decrease in blood pressure (BP)] that reproduced a patient’s spontaneous symptoms of recurrent transient loss of consciousness with spontaneous recovery.

### Protocol for the head-up tilt test

The protocol used for tilt-table testing has been described elsewhere^1,3–8^ and essentially consisted of a 70° baseline upright tilt for a period of 30 minutes, during which time, the heart rate and BP were monitored continually. If no symptoms occurred, the patient was lowered into the supine position and an intravenous infusion of isoproterenol was started with a dose sufficient to raise the heart rate to 20% to 25% above the resting value. The upright tilt was then repeated for a period of 15 minutes.

### Criterion for diagnosing combined orthostatic intolerance

Patients were included in this study if they reported clinical symptoms consistent with both POTS and NCS and demonstrated a typical POTS pattern (ie, a rise in heart rate without a change in BP) upon assuming an upright posture or during HUTT within the first 10 minutes of upright tilt followed by a neurocardiogenic pattern with continued HUTT (ie, a sudden fall in heart rate and/or fall in BP), reproducing symptoms that were similar to their spontaneous episodes.^1^

### Protocol for medication use

The treatment protocols employed were based on previous experiences with orthostatic disorders and are described in detail elsewhere.^3–9^ Briefly, a sequence of therapies was employed that included physical counter maneuvers and aerobic and resistance training as well as increased intake of dietary fluids and sodium. If these methods were ineffective, pharmacotherapy was initiated in a sequence generally consisting of β-blockers, central sympatholytic drugs, fludrocortisone, midodrine, and selective serotonin reuptake inhibitors, either alone or in combination with one another.

If patients failed to respond to these medications, second- and third-line medications like octreotide, erythropoietin, and pyridostigmine were employed. As this was a retrospective chart review study, neither a formal survey by questionnaire to assess patients’ response to treatment nor an assessment of their response to treatment by HUTT was conducted. Information about the subjective symptoms and sense of well-being of each patient was collected from the patient charts, through communications with physicians, and by direct patient inquiry. A treatment was considered successful if the patient reported that it provided symptomatic relief.

### Loop recorder insertion

Patients underwent the insertion of a loop recorder (Reveal ILR; Medtronic, Minneapolis, MN, USA). We have previously published our experience with the technique and the details of follow-up of patients with loop recorders.^10^

Patients were followed up with in our clinic every three to six months and after both automatic and patient-activated transmissions had occurred. If patients demonstrated a pause of more than three seconds with syncope or presyncope or a pause of more than six seconds without syncope, they underwent dual-chamber pacemaker implantation.

### Statistics

This was an observational study. The statistical analysis was performed using the Statistical Package for the Social Sciences version 17 software program (IBM Corporation, Armonk, NY, USA). Continuous data are presented as mean ± standard deviation values and categorical data are presented as percentages. A t-test was used for the comparison of mean values, with statistical significance reached at a p-value of less than 0.05.

## Results

A total of 500 charts of patients were screened and followed up with at the University of Toledo autonomic center clinic. These patients had been seen over a period of 10 years. We found 40 patients, with a mean age of 33 ± 13 years, including 32 (80%) women, who met the inclusion criterion for this study **(Table 1)**. This group of patients was diagnosed with POTS based on their clinical characteristics and/or responses on HUTT. All these patients had unusually frequent symptoms of presyncope and syncope and had experienced at least three to four episodes of syncope within six months. The other clinical characteristics of these patients are summarized in **Table 1**. To better understand the nature and mechanism of their syncope, all 40 patients underwent loop recorder implantation with a Reveal XT monitor (Medtronic, Minneapolis, MN, USA). These patients were followed up with at our syncope clinic on a regular basis.

All study participants had failed first-line medications. Second-line medications, including pyridostigmine and octreotide, and fluid therapy were trialed but without any benefit.

Forty patients demonstrated prolonged asystole (> 6 seconds) or severe bradycardia (heart rate < 30 bpm) during their syncope. Ten patients demonstrated an asystole of more than 10 seconds and also had prolonged convulsive syncope. All patients had abrupt syncope without any warning signs. All patients underwent dual-chamber pacemaker implantation using a closed-loop stimulation algorithm (Biotronik, Berlin, Germany). Syncope was eliminated in all patients following pacemaker implantation; however, they continued to experience orthostatic tachycardia.

## Discussion

POTS is a group of various disorders that share a similar physiological state.^11–18^ Syncope is rare in POTS and has been reported in 10% to 38%^19^ of patients in general but occurred in all patients in this highly selected group of our study. Syncope in POTS could be explained by a late-phase surge in parasympathetic tone or sympathetic withdrawal, leading both to cardioinhibition and vasodepression.^1,2,20^ A combined form of OI has been previously reported.^1^ These patients demonstrate a typical POTS pattern (ie, a rise in heart rate without a change in BP) upon assuming an upright posture or during HUTT within the first 10 minutes, followed by a neurocardiogenic pattern with continued HUTT, demonstrating a sudden fall in heart rate and/or fall in BP, ultimately reproducing symptoms that were similar to the patients’ spontaneous episodes.

All patients in this study had difficulty in managing their POTS and experienced unusually frequent syncope episodes. Each patient failed multiple trials of various medications. ILR insertion in these patients previously revealed that they may have a prolonged mechanism of their syncope and, to rule out any arrhythmic etiology, all patients underwent loop recorder insertion.^2^ All patients in this study were found to have prolonged episodes of asystole or complete heart block (6–10 seconds) and subsequently underwent dual-chamber pacemaker implantation.

It is important for physicians and patients to understand that not every patient with POTS may benefit from a pacemaker; instead, only a subgroup of patients with recurrent syncope and documented asystole on cardiac monitoring may. Although syncope was eliminated in all patients who received a dual-chamber pacemaker, most of the patients continued to have orthostatic tachycardia and dizziness. Thus, another important concept to understand is that pacemakers cannot improve symptoms of OI like palpitations and dizziness. It is also important to document asystole or bradycardia in POTS patients before offering them a pacemaker. All POTS patients who present with unusually frequent and severe episodes of syncope are evaluated using an ILR at our center. Prolonged monitoring in such patients helps establish a better symptom–rhythm correlation in this subgroup of patients and may also help in selecting those patients who may benefit from subsequent pacemaker implantation. Patients with POTS are not immune to other causes of syncope like both brady- and tachyarrhythmias. Prolonged monitoring in such patients may reveal an underlying etiology and may lead to proper management and improvement of the quality of life in those who suffer from disabling syncope.

### Limitations

There were several limitations in the current study that are important to consider. First, this study was retrospective and included a small number of patients, none of whom underwent additional autonomic function assessment besides HUTT. Also, we did not deploy a quality-of-life assessment questionnaire in this group of patients. While interpreting the results of this small study, we want both physicians and patients to understand that pacemaker therapy may benefit only a small subset of POTS patients who demonstrate prolonged asystole on a cardiac monitor. Also, a pacemaker may not improve other symptoms of OI and may help only with syncope.

## Conclusion

Dual-chamber pacing helps to eliminate syncope in a subgroup of POTS patients with recurrent syncope and prolonged asystole on ILR. However, these patients will continue to have symptoms of orthostatic tachycardia.

## Figures and Tables

**Table 1:** Baseline Clinical Characteristics of the Study Participants (n = 40)

Age (years)	33 ± 13
Sex (female)	32 (80%)
Symptoms of OI	
Orthostatic palpitations	36 (90%)
Dizziness	36 (90%)
Inability to concentrate	32 (80%)
Syncope	40 (100%)
Presyncope	40 (100%)
Fatigue	36 (91%)
Chest pain	24 (60%)
Medications	
β-blocker	20 (50%)
SSRI	18 (45%)
NERI/SSRI	24 (61%)
Midodrine	20 (50%)
Modafinil	7 (18%)
Fludrocortisone	33 (33%)
Pyridostigmine	32 (80%)
Octreotide	2 (6%)
Erythropoietin	13 (33%)
Comorbid conditions	
Hypermobility	8 (20%)
HTN	2 (6%)
DM	1 (3%)
Migraine	20 (50%)
Precipitating factors for POTS	
None	27 (66.6%)
Infectious mononucleosis	7 (17%)

DM: diabetes mellitus; HTN: hypertension; NERI: norepinephrine reuptake inhibitor; OI: orthostatic intolerance; POTS: postural orthostatic tachycardia syndrome; SSRI: selective serotonin reuptake inhibitor.
